# Effects of Tricaine Methanesulphonate (MS-222) on Physiological Stress and Fresh Quality of Sea Bass (*Lateolabrax maculatus*) under Simulated High-Density and Long-Distance Transport Stress

**DOI:** 10.3390/biology12020223

**Published:** 2023-01-30

**Authors:** Hongzhi Zhang, Qi Wang, Yixuan Dong, Jun Mei, Jing Xie

**Affiliations:** 1 College of Food Science and Technology, Shanghai Ocean University, Shanghai 201306, China; 2 National Experimental Teaching Demonstration Center for Food Science and Engineering, Shanghai Ocean University, Shanghai 201306, China; 3 Shanghai Engineering Research Center of Aquatic Product Processing and Preservation, Shanghai 201306, China; 4 Shanghai Professional Technology Service Platform on Cold Chain Equipment Performance and Energy Saving Evaluation, Shanghai 201306, China

**Keywords:** MS-222, transport density of live fish, stress, nutrition, flavor

## Abstract

**Simple Summary:**

The traditional method of transporting live fish in water often involves long driving durations and a high transport density. These conditions not only cause the deterioration of transport water quality but also induce stress in fish, seriously affecting their physiological and metabolic responses. At the same time, it also causes changes in the flavor of fish muscle. In this study, methanesulphonate (MS-222), a common anesthetic, was applied to live fish during transportation in order to slow down the deterioration of the water environment quality, reduce the stress response of sea bass, and improve meat flavor. The effects of different transport densities on water quality, oxidative stress, energy metabolism, and meat flavor of sea bass were measured under the conditions of adding MS-222 concentration of 30 mg/L and different transport densities for 72 h, and these effects were compared with those without MS-222. The results showed that the water quality, oxidative stress, and energy metabolism of anesthetized fish were lower than those of the control group at the same density. The addition of 30 mg/L MS-222 to stabilize fish under high density transportation can reduce the deterioration of water quality and the effect on the stress of live fish, and improve meat flavor.

**Abstract:**

This study aimed to evaluate the effect of different transport densities on water deterioration, physiological response, nutrients, and fresh quality of sea bass (*Lateolabrax maculatus*) at 30 mg/L tricaine methanesulphonate (MS-222) before and after simulated live transport. The results indicated that the addition of MS-222 could effectively decrease mortality compared with the control (CK) sample during the simulated live transport. The concentration of dissolved oxygen was lower and the total ammonia nitrogen was higher in the high transport density samples than those of low transport density samples after 72 h in transport. The level of blood cortisol (COR), glucose (GLU), lactic acid (LD), aspartate aminotransferase (AST), alanine aminotransferase (ALT) for the sea bass were significantly higher compared with the CK sample (*p* < 0.05) during the simulated live transport and after 12 h of recovery. These results indicated that the sea bass presented a strong stress response in high-density transport. The glycogen, fat, and protein of the sea bass were degraded to supply the energy for the body in the process of surviving the transportation, resulting in the decreased nutrient content in the muscle, which recovered to the initial level (CK) after 12 h. The increase in flavor substance content, such as free amino acids, nucleotides, organic acids, and minerals, enhanced the special flavor of the muscle during the simulated live transport. This study demonstrates that the addition of MS-222 at 30 mg/L to the transport water is an effective method for live fish to realize low mortality and physiological response during high-density and long-distance transport.

## 1. Introduction

For the past few years, the yield of marine fish showed a continuous downward trend, and cultured marine fish showed a continuous upward trend in China, reaching 1.844 million tons in 2021 [1]. Sea bass (*Lateolabrax maculatus*) is widely cultured, and the production has reached more than 199,000 tons in China [1,2]. Fresh sea bass, after slaughter, is prone to being perishable in the process of transportation and refrigerated storage [2,3]. It is difficult to buy live sea bass on the market due to the high mortality of live fish during its transport [4,5]. Therefore, research on live fish transportation is essential.

High-density and long-distance transportation can lead to high stress in live fish. Anesthesia is required to sedate fish, and it promotes fish welfare [6]. As shown in Table 1, several anesthetics commonly used to anesthetize fish include tricaine methanesulfonate (MS-222), clove oil, 2-phenoxyethanol, and carbon dioxide (CO_2_). Until now, only MS-222 has been approved by the U.S. Food and Drug Administration (USFDA) for extensive use in live fish, and the fish is allowed to be sold on the market after 21 days of temporary culturing [7]. However, in the United Kingdom, Canada, Italy, Spain, and Norway, only registered veterinarians are permitted to administer MS-222 to fish. There is a lack of similar regulations for MS-222 used in aquatic animals in China [8]. An appropriate amount of MS-222 slows down the breathing and movement of fish, thus reducing metabolism and oxygen consumption; fish enters a dormant state, which is suitable for long-distance transportation. The mechanism of MS-222 is as follows: absorption of MS-222 temporarily blocks the sensory (afferent) nerve conduction in live fish to restrain activity and reflex ability and relieve their susceptibility, pain, discomfort, struggle, and physical energy consumption induced by transport stressors [9,10,11]. MS-222 mainly accumulates in the spleen, liver, and other organs, but its content in the muscle is very small, and it is easy to transfer from aquatic products to water in clear environments. Because it has the advantages of quick effect, short recovery time, and high safety, MS-222 has been widely used in fish and other aquatic creatures during their handling and transportation [8]. Rozynski et al. [10] found that there was no difference in glucose (GLU), total protein (TP), and lactate (LD) values of pikeperch (*Sander lucioperca*) exposed to MS-222 aqueous solution after 24 h compared with the initial sample. Zhao et al. [12] showed that the use of MS-222 with temperature reduction for *Coreius guichenoti* plays a key role in decreasing the content of cortisol, lactate, and transaminase activity in serum, as well as improving water quality, which indicates that MS-222 treatment might cause a reduction in the metabolism and in the energy demand of fish through the alleviation of stress effect [13]. Hong et al. [14] studied the effect of water quality and stress response for golden pompano (*Trachinotus ovatus*) transported in different densities. The physiological stress indexes showed an upward trend, while liver parameters and water quality showed a downward trend. These studies indicated that the application of anesthetics could efficiently reduce the physiological stress responses of organisms and the deterioration of the transportation water during live fish transport.

Transportation and handling not only affect and change the physiological mechanism of live fish but also affect nutrition and meat quality. Flavor and nutrition are crucial factors in improving the fish quality while preserving the vitality of live fish is a major challenge in the transportation and marketing of live fish [15]. Cao et al. [16] studied the effects of adding MS-222 to the transportation water on the nutrition, flavor, and water quality of turbot; they found that water quality, texture characteristics, and water, fat, and protein in fish muscle showed a decreasing trend, while fresh bitter amino acids increased.

Wu et al. [17] studied the effects of ascorbic acid and β-1, 3-glucan on the stress response and muscle quality of tiger grouper after simulated transport and found that tiger grouper had a high survival rate, low serum cortisol, and HSP70 content and no deterioration in flavor and nutritional indicators. Espinoza-Ramos et al. [18] studied the effect of transportation time and stocking density on the survival of Peruvian grunts and found that changes in seawater quality play a decisive role. In the present, although the effects of the fish stress response, physiological regulation, and fresh quality have been reported during live fish transportation [19,20], few studies on high-density transportation have been conducted. Live fish should be transported more efficiently under high-density conditions [21]. Therefore, the aim of this study was to explore the maximum transport density, physiological response, nutrients, and fresh quality of sea bass with the addition of 30 mg/L MS-222 to the transport water before and after the simulated live transport.

**Table 1:** Several anesthetics commonly used in fish and their characteristics.

Anesthetic	Characteristics	References
Tricaine Methanesulphonate (MS-222)	Anesthetic with analgesic effects. Fast induction and recovery Available as a powder to be dissolved in water. There is no enrichment, and there is less residue in the muscle.	Liu et al. [8]
Clove Oil	Low cost Little impact on human health and environment. It is volatile and its efficiency decreases with the prolongation of transportation time. Affect the muscle flavor of fish. A slight difference in dose used may result in fish death or a slow recovery time for surviving fish.	Kamble et al. [22]
2-Phenoxyethanol (C_8_H_10_O_2_)	Low cost Colorless, oily, aromatic liquid. Easy to prepare and soluble in water. Rapid effect and recovery time. It can kill bacteria and fungi. Slight changes in the internal organs and tissues of the fish.	Priborsky et al. [23]
CO_2_	Low cost No residue in the body, no pollution of the environment. Slow induction and recovery. The dosage is difficult to control, the anesthetic effect is unstable, and the scope of application is narrow.	Oberg et al. [24]

## 2. Materials and Methods

### 2.1. Preparation of Sea Bass

Sea bass (500 ± 50 g, 39 ± 1 cm) was purchased from the local market in the town of Luchao Port (Shanghai, China) and transported to the laboratory in a live fish transport box. Sea bass of uniform size with uniform respiratory rates was deprived of food in a temporary pond for 36 h; the water was continuously inflated to maintain the dissolved oxygen content during the period of temporary culture. The parameters of the temporary culture were as follows: the fish to water ratio was 1:50, the water temperature was 20~22 °C, the salinity was 16‰, the dissolved oxygen was 4~6 mg/L, and the pH was 7.5~8.5.

### 2.2. Experimental Design

**Figure 1:**
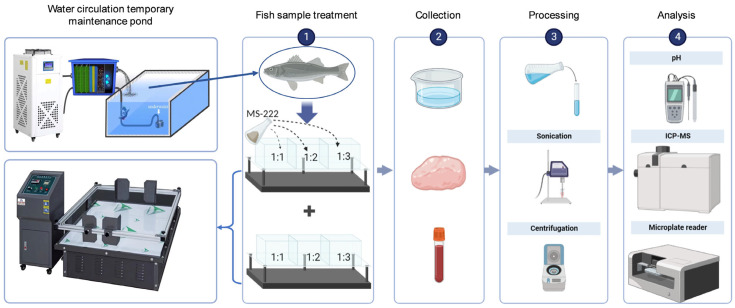
Schematic diagram of experimental design for simulating long-distance transportation of sea bass.

The results of the pre-experiment allowed us to determine the best concentration of MS-222 (30 mg/L), which can make the sea bass achieve deep anesthetic effect. After temporary cultivation, the temperature was decreased from 20~22 °C to 12 °C at a rate of 3 °C/h. The other parameters of the transport water were the same as those of the cultured water. The sea bass was treated with 30 mg/L MS-222 and divided into three treatment samples: the transport density was 1000 g/L (fish to water ratio was 1:1, MS-1-1), 500 g/L (fish to water ratio was 1:2, MS-1-2), and 333.3 g/L (fish to water ratio was 1:3, MS-1-3), respectively. The sea bass was not treated with MS-222 and divided into three treatment samples: the transport density was 1000 g/L (fish to water ratio was 1:1, NMS-1-1), 500 g/L (fish to water ratio was 1:2, NMS-1-2), and 333.3 g/L (fish to water ratio was 1:3, NMS-1-3), respectively. The samples of sea bass without transport or addition of MS-222 were used as control (CK). Then, the fish was stocked in a live fish transport box with cold water (12 °C) and subjected to simulated transport for 72 h. The number of fish used in each sample was 30. The simulated transport was listed as follows: 1 h on a B-level road (80 km/h) → 4 h on an A-level road (100 km/h) → 1 h on a B-level road (80 km/h) and repeated 12 times. Five sea bass were randomly selected for sampling after being transported for 72 h, and the sea bass samples were recovered for 12 h in the cultured water at room temperature and selected for sampling. The survival rate of the animals in each sample was determined [25,26]. The experimental design process is shown in Figure 1.

$$Survival\;rate=\frac{Number\;of\;surviving\;fish}{Number\;of\;fish\;in\;sample}\times 100\%$$

### 2.3. Pre-Treatment of Samples

The sea bass was stunned with ice for 15 min and then killed in accordance with the principles and guidelines established by the Animal Care and Use Committee of Shanghai Ocean University (SHOU-DW-2021-67). The blood of sea bass was taken from the tail vein without anticoagulant. The blood was stored at 4 °C for 2 h, then centrifuged at 10,614× *g*, 4 °C for 5 min, and the liquid supernatant (serum) was collected. The serum was stored at −80 °C before using for the index determination. Note: The serum should not be used after repeated freezing and thawing. The back muscles of the sea bass were taken with a scalpel on an ice board, then rinsed with normal saline, and stored in a refrigerator at −80 °C. The physiological indexes and nutritional flavor indexes of the muscles were determined.

### 2.4. Determination of Indicators

#### 2.4.1. Determination of Water Quality Indicators

At each test point of simulated transportation, 100 mL of transport water was taken with a centrifugal tube to measure water quality indicators. According to the method of Ma et al. [27], pH value is measured by using a pH meter (Sartorius, PB-10). The total ammonia nitrogen concentration (mg/L) was determined by reagent colorimetry (Kyoritsu Chemical-Check); dissolved oxygen concentration (mg/L) was measured by using a dissolved oxygen meter (JPSJ-605F, Yi Electrical Scientific Instrument Co., Ltd., Shanghai, China).

#### 2.4.2. Determination of Biochemical Blood Indicators

The detection method of cortisol (COR) was an enzyme-linked immunosorbent assay (ELISA). Commercial fish ELISA kits for COR were supplied by Jiancheng Bioengineering Institute (Nanjing, China). Glucose (GLU), lactic acid (LD), alkaline phosphatase (ALP), aspartate aminotransferase (AST), and alanine aminotransferase (ALT) in the serum were measured using commercial kits (Jiancheng Bioengineering Institute, Nanjing, China). After samples were processed according to the operating instructions of the kit, the OD value was read with a spectrophotometer (Eppendorf, Biophotometer RS-232, Hamburg, Germany), and the content of stress indicators in the serum was calculated [28,29].

#### 2.4.3. Determination of Nutrients in Fish

Water content was determined using a moisture meter (Shanghai Hutong Industrial Co., Ltd. HX-Q10, Shanghai, China).

Glycogen (Gly) and total protein (TP) in the muscle were measured spectrophotometrically using commercial kits (Jiancheng Bioengineering Institute, Nanjing, China).

The crude fat content was measured by the Soxhlet extraction method, 5 g samples of fish (*M*_1_), and the mass of the bottom bottle (*M*_2_) were accurately weighed. The samples were wrapped with filter paper and placed in the bottom bottle for extraction with an automatic cable fat extraction instrument (FOSS Soxtec 2050, Coppenhagen, Denmark). After extraction, the bottom bottle was dried in a drying oven at 103 °C for 1 h. The bottom bottle was removed and placed in a dryer to cool and be weighed (*M*_3_). The calculation method was as follows:

$$Crude\;fat\;content=\frac{M_{3}-M_{2}}{M_{1}}$$

#### 2.4.4. Determination of Flavor Substances in Fish

FAAs were determined as described by Pei et al. [30] using an amino acid analyzer (Hitachi L-8800, Tokyo, Japan). The FAAs identification and quantification were completed using the retention time and peak area with reference to FAAs standards (Sigma Chemical Co., St Louis, MO, USA). The sum of each free amino acid detected in the sample was the total amino acid content.

The ATP-related compounds were measured by HPLC (Waters 2695, Milford, CT, USA), according to Cao et al. [31].

After digestion, the content of inorganic salt ions was determined by an inductively coupled plasma mass spectrometer (Thermo Fisher, ICP-MS, Waltham, MA, USA) [32,33]. After extraction, the samples were purified by a strong anion exchange solid phase extraction column and separated by a reversed-phase chromatography column, followed by qualitative assessment using retention time and quantitative assessment using the standard external method. The sample solution was injected into the high-performance liquid chromatography (Thermo, U3000) to obtain the peak height or peak area, and the concentration of organic acids in the solution to be measured was obtained according to the standard curve [34].

## 3. Statistical Analyses

The one-way ANOVA-Duncan test program in SPSS 26.0 software was used for multiple comparisons. The Levene test was used to check the homogeneity of the samples before applying Duncan. The results were expressed as means ± SD, and the significance threshold was 0.05. Origin software was used to make graphs.

## 4. Results and Discussion

### 4.1. Survival Rate

**Figure 2:**
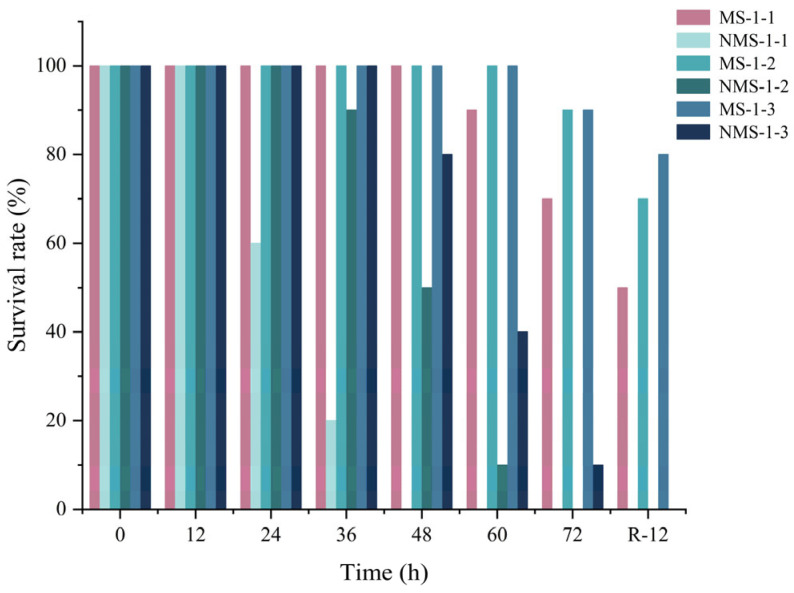
The survival rate of sea bass in treatment samples and basal samples during and after transport. MS-1-1: 30 mg/L MS-222, fish to water ratio was 1:1; NMS-1-1; fish to water ratio was 1:1; MS-1-2: 30 mg/L MS-222, fish to water ratio was 1:2; NMS-1-2: fish to water ratio was 1:2; MS-1-3: 30 mg/L MS-222, fish to water ratio was 1:3.; NMS-1-3: fish to water ratio was 1:3.

The survival rates of sea bass in differently treated samples during the simulated live transport are presented in Figure 2. The survival rates of sea bass decreased with the transport density expansion, which was attributed to the rapid consumption of the dissolved oxygen and the high concentration of ammonia excretion in the water during the simulated live transport [35]. The survival rate of non-MS-222-treated samples was distinctly lower than the MS-222-treated samples at the same transport density, indicating that the transport of water with MS-222 could effectively reduce the mortality of live fish. The survival rate of MS-1-2 samples was in accordance with the MS-1-3 samples after 72 h transport (90%). The survival rate of MS-1-2 samples was lower than that of the MS-1-3 samples after recovery of 12 h as the live sea bass suffered irreparable damage caused by the gradually deteriorating environment and mechanical oscillation during high-density transportation.

### 4.2. Water Quality Indicators

**Figure 3:**
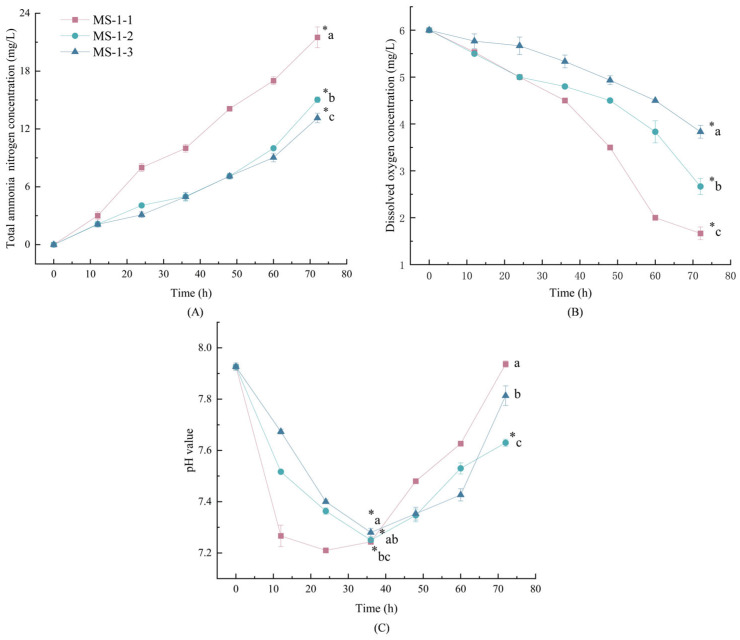
The changes in total ammonia nitrogen concentration (**A**), dissolved oxygen concentration (**B**), and pH value of transport water (**C**) in different treatment samples during live transport. Vertical bars indicate standard deviation. Asterisk (*) represents significant differences (*p* < 0.05) at transporting for 0 h and after transport. Different lowercase letters represent significant differences among three treatment samples at the same time during live fish transport.

The respiration and excretion accumulation of fish caused a substantial raise in total ammonia nitrogen concentration and a decrease in the dissolved oxygen level along with the transport time [16]. High transport density led to rapid deterioration of the water quality. There were significant differences among the three MS-222 treatments after transport for 72 h compared with the ammonia concentration of no-transport samples (*p* < 0.05) (Figure 3A). The highest concentration of ammonia was in MS-1-1 samples, which gradually increased from 0 mg/L up to 21.5 mg/L after 72 h transport; the maximum values of ammonia for MS-1-2 and MS-1-3 were 15.03 mg/L and 13.13 mg/L, respectively. This is consistent with what Franklin and Edward said [36]; a directly proportional relationship was obtained between ammonia concentrations and high densities, with the degradation of seawater quality because of the increase in fish excretion metabolites. During the simulated transport of live fish, the dissolved oxygen in the transport water of the three MS-222-treated samples showed a decreasing trend, which was significantly different from the samples at 0 h (*p* < 0.05) (Figure 3B). The rank of the dissolved oxygen consumption for three MS-222-treated samples in order from high to low was MS-1-1, MS-1-2, and MS-1-3. There were significant differences among the three samples (*p* < 0.05). The pH value of transport water initially decreased and then increased during the simulated transport of live fish (Figure 3C). The pH value reached a minimum value at the 36th h, and then increased with the transport time, due to the increase in carbon dioxide concentration during the respiration and metabolism of animals, which lead to the decrease in pH at the early transport time. Then, the accumulation of ammonia excretion by fish led to the alkalinity of the transport water [37,38,39].

### 4.3. Blood Biochemical Indicators

The levels of cortisol, glucose, and lactate in the serum are low under normal circumstances. When the fish is affected by stress, the hormones are released, blood sugar increases to maintain the body’s normal life activities, and lactic acid increases to cause anaerobic respiration [17,38]. The activity of ALT, AST, and ALP are stable and low under normal conditions; however, when the fish is subjected to external stress resulting in liver and other tissue damage, the activation of the transaminase activity in the body increases or decreases to varying degrees [40]. The levels of COR, GLU, LD, and the activities of AST and ALT showed a rising trend in all samples after transport for 72 h. The activity of ALP showed a downtrend (Table 2). Some studies have indicated that the stress response of fish was severe in the initial stage of transport; these reactions were alleviated by the self-recovery of fish along with the increase in transport time. The fish died as the pressure exceeded a threshold [14]. The concentration of COR increased in each MS-treated sample with a significant difference among MS-1-1, MS-1-2, and CK samples after being in transport for 72 h (*p* < 0.05), but there was no difference in COR level of MS-1-3 samples compared with that in the CK sample (*p* > 0.05). Some studies have shown that anesthetics affect the release of serum cortisol in fish [41]. However, the transport density here has a greater effect on fish stress. Espinoza-Ramos et al. found that transport density affects the change in seawater quality, resulting in the increase in nitrogen compounds and a decrease in pH value, which leads to the increase in stress response in fish, which has an adverse effect on the viability of fish during transportation [18].

After anesthesia, the fish suffer less stress and fewer physiological changes at lower transport densities. In addition, individual differences cannot be ignored [42]. It was found that the levels of COR, GLU, LD, AST, and ALP in the MS-1-1 sample were obviously higher than those of the MS-1-3 sample after being in transport for 72 h (*p* < 0.05). After 72 h transportation, there was no difference in alanine aminotransferase activity among the three MS-222-treated samples (*p* > 0.05). Boaventura et al. [29] also reported that respiration and metabolism under high-density conditions were higher than those under low-density conditions during the simulated transport. The concentrations of GLU and LD and the activity of AST in the three MS-222-treated samples were obviously higher than those of CK. The utilization rate for GLU and metabolism of sea bass at low temperatures were low, and the release of COR led to increased glutamate levels in the blood [43]. According to Liu et al. [8], MS-222 treatment resulted in lower serum cortisol levels and higher serum glucose content in yellow catfish. Similar findings were also reported in juvenile silver pomfret (*P. argenteus*) after being exposed to MS-222 treatment [44]. The sea bass breathed without oxygen to resist transport stress, which caused the tissue to be damaged during the simulated transport, so it needed time to recover [45].

ALT and COR activities returned to the level of the CK samples, and no differences were observed in COR and ALT among CK, MS-1-2, and MS-1-3 samples after 12 h of recovery. However, the COR level in the MS-1-1 sample did not return to the level of the CK samples. The decrease in the hormone levels and transaminase activity indicated that the stress responses and adverse effects of fish were gradually eliminated by this study date; however, the samples in the higher transport density sample (MS-1-1 sample) needed time to recover thoroughly. The levels of GLU, LD, AST, ALT, and ALP of MS-1-2 samples were lower than those of MS-1-1 samples after 12 h of recovery (*p* < 0.05). AST and ALT in MS-1-1 samples had the highest values; AST and ALT via gluconeogenesis caused the glucose increase in the organism [46], and the stress still existed in the MS-1-1 samples after recovery for 12 h. The high-density and long-time transport of live fish induced irreparable injury for sea bass.

### 4.4. Basic Nutrients

There were no changes observed in the moisture content of sea bass muscle before and after transport (*p* > 0.05, Table 3). Crude fat, total protein, and muscle glycogen content of the sea bass were low compared with those of the CK samples after transportation for 72 h (*p* < 0.05), which is ascribed to the sea bass gradually consuming glycogen to maintain the balance of energy metabolism. Nutrients, such as crude fat and protein, are broken down to meet the energy supply, resulting in a reduction of nutrients in sea bass [47]. There were significant differences in muscle glycogen concentration among the three MS-222-treated samples after 72 h in transport (*p* < 0.05), and no differences were found in crude fat and total protein between MS-1-2 and MS-1-3 samples (*p* > 0.05) after 72 h in transport. The crude fat, total protein, and muscle glycogen content of the three MS-222-treated samples presented an uptrend after 12 h of recovery resulting from hunger during and after transportation.

### 4.5. Nitrogenous Flavor Compounds: Free Amino Acids and Nucleotides

Free amino acids are the main taste substances of fish, such as umami, sweetness, and bitterness. Asp and Glu provide a umami taste; Thr, Ser, Gly and Ala provide a sweet taste; and Val, Met, Ile, Leu, Tyr, Phe, Lys, His, and Arg provide a bitter taste [48,49]. The changes in the content of free amino acids and nucleotides of sea bass during and after the transport of live fish are shown in Table 4. Fifteen free amino acids were separated and detected. The total content of free amino acids in sea bass was 349.71 mg/kg in the CK samples. The most abundant free amino acid is Gly (95.02 mg/kg), followed by Ala (78.41 mg/kg), Lys (55.89 mg/kg), and Glu (31.24 mg/kg). These four free amino acids are the main flavor components.

After 72 h of the simulated live transport, the total amount of free amino acids increased, and MS-1-2 and MS-1-3 samples returned to the level of the CK samples, which was similar to those of Wu et al. [17]. Since there was no feed for sea bass during the simulated live transport, the protein in fish is degraded into an energy source for fish consumption, resulting in a decrease in nutrient content and an increase in free amino acids [5]. The content of free amino acids in sea bass during high-density transport was higher than during low-density transport.

Umami amino acids in MS-222-treated samples increased by 6.70%, 7.28%, and 4.00% compared to the no-MS-222-treated samples after 72 h of the simulated transport, respectively. The sweet amino acids and bitter amino acids in MS-222-treated samples increased. The stress effects and energy respiration metabolism of samples were stronger in the higher-density transport sample than in the other samples, and the high amino acid content was caused by the acceleration of protein degradation [14,50]. The result of free amino acid was consistent with the results of water quality deterioration, stress response, and lipid and protein energy consumption, which indicated that the rate of amino acid increase after the transport of live fish was positively correlated with the density of the transport [51]. Free amino acids gradually recovered to the content of the CK samples after 12 h of recovery. The metabolic processes of amino acids are extremely complicated. However, there are few studies on the amino acid metabolism of fish under stress. The changes in different amino acids after stress may be related to the difference in fish species, stress time, and starvation time [52]. The simulated live transport of sea bass in this study can help to understand the effects of stress on fish flavor.

The nucleotide is the key parameter to evaluate the flavor of fish. IMP imparts a meaty and sweet flavor contributing to improving the quality of fish, whereas its transformation to HxR and Hx results in unpleasant bitterness [53,54,55,56]. The changes in the nucleotide of the sea bass before and after the simulated live transport were shown in Table 4. IMP increased, and AMP decreased in each sample after 72 h transport and recovered to the level of the CK samples after 12 h with no significant difference (*p* > 0.05). Cao et al. [57] also found that with the extension of transportation time, the AMP content of the turbot first decreased and then increased. After 18 h, AMP was still at a high level, indicating that the low-temperature transportation operation could increase the fresh flavor-presenting substances of turbot and improve the flavor of fish flesh. The increase in IMP caused by the breakdown of ATP and the degradation of nucleotides gave rise to the decrease in AMP. However, Wu et al. [17] found that the content of IMP in tiger samples decreased after the transport and then recovered to the initial levels, indicating that the effect of transport on nucleotide degradation in the muscle of sea bass could be recovered.

### 4.6. Non-Nitrogenous Flavor Compounds: Organic Acids and Inorganic Salts Ions

Organic acids are the essential taste substances in fish, and the common organic acids include acetic acid, lactic acid, succinic acid, tartaric acid, malic acid, etc. [58]. However, only three organic acids were detected in the muscle of sea bass: lactic acid, acetic acid, and fumaric acid. The changes in the organic acids of sea bass are shown in Table 5. The content of lactic acid in the muscle of sea bass accounted for the highest percentage of organic acids. Lactic acid, acetic acid, and fumaric acid in the sea bass increased during the simulated transport and decreased after the recovery. The MS-222-treated samples had higher organic acids content than that of the CK samples, and higher transport density had higher organic acids content. This could be due to the metabolic intensity, and physiological responses of the body of perch being larger in the high-density transport samples with strong stress response, resulting in the accumulation of organic acids in muscle [14,59]. At present, there are few reports on the effects of live transport on organic acids in fish muscle, and this study provided a reference for the flavor of organic acids after the transport of live fish.

Minerals play an important role in the life-sustaining activity and physiological accommodation of aquatic products [60]. The essentiality of macro-minerals (Ca, P, Mg, Na, and K) and certain trace elements (Cu, Fe, and Zn) have been determined in fish, which could enhance the characteristic taste of fish [61,62,63]. The minerals in the sea bass muscle showed different degrees of changes after 72 h of simulated transportation. However, there were no significant differences in Mg, P, Ca, Fe, Cu, and Zn in all samples before and after transport (*p* > 0.05).

Na and K in MS-222-treated samples increased with a significant difference after transportation for 72 h compared with the no-MS-222-treated samples (*p* < 0.05) and recovered to the initial level (CK) after 12 h of recovery. Mineral content in fish is related to the metabolic level, and osmotic regulation of the body, as well as Na^+^ and K^+^, has a salty taste [64,65,66]. The increase in fish salinity indirectly indicated that transportation could affect the response of ion osmotic regulation of perch, and the difference between individuals should not be ignored.

## 5. Conclusions

Our data suggest that the addition of MS-222 can effectively reduce mortality during high-density transportation in vivo. In the simulation of long-distance live transport, the addition of MS-222 30 mg/L is helpful to transport sea bass under the condition of maximum density upper limit of 500 g/L (fish to water ratio is 1:2) for 72 h. It can reduce the stress pressure on sea bass by improving water quality and reducing the changes in stress response indexes such as COR. In addition, the increase in free amino acids, nucleotides, organic acids, and minerals enhanced the special flavor of muscle during transportation. The energy metabolism of fish due to stress can return to the initial level after 12 h of recovery.

## Figures and Tables

**Table 2:** Effects in blood biochemical stress of the sea bass before and after simulated live transport.

Indicators	Samples	Simulated Transport Time (h)		
		0 (CK)	72	Recover-12
Cortisol (COR) (ng/L)	MS-1-1	178.37 ± 4.67 ^a^	264.20 ± 9.48 ^Ab^	227.54 ± 9.42 ^Ab^
	MS-1-2		240.87 ± 8.90 ^Ab^	224.62 ± 7.13 ^Aab^
	MS-1-3		201.29 ± 2.59 ^Ba^	189.62 ± 3.53 ^Aa^
Glucose (GLU) (mmoL/L)	MS-1-1	1.48 ± 0.10 ^a^	8.34 ± 0.39 ^Ab^	3.46 ± 0.09 ^Ac^
	MS-1-2		4.29 ± 0.02 ^Bb^	2.69 ± 0.02 ^Bc^
	MS-1-3		3.54 ± 0.14 ^Cb^	1.95 ± 0.19 ^Cc^
Lactic acid (LD) (mmoL/L)	MS-1-1	1.65 ± 0.01 ^a^	3.24 ± 0.00 ^Ab^	1.93 ± 0.04 ^Ac^
	MS-1-2		3.23 ± 0.04 ^Ab^	1.83 ± 0.01 ^Bc^
	MS-1-3		3.75 ± 0.12 ^Bb^	2.03 ± 0.04 ^Ac^
Aspartate aminotransferase (AST) (U/L)	MS-1-1	94.23 ± 1.27 ^a^	198.44 ± 2.71 ^Ab^	123.50 ± 2.93 ^Ac^
	MS-1-2		176.86 ± 6.43 ^Bb^	107.71 ± 2.91 ^Bc^
	MS-1-3		171.71 ± 2.35 ^Bb^	110.01 ± 4.44 ^Bc^
Alanine aminotransferase (ALT) (U/L)	MS-1-1	48.92 ± 1.55 ^a^	85.34 ± 2.42 ^Ab^	67.08 ± 2.54 ^Ac^
	MS-1-2		80.35 ± 2.84 ^Ab^	54.36 ± 2.64 ^Ba^
	MS-1-3		74.09 ± 13.07 ^Ab^	51.10 ± 2.67 ^Ba^
Alkaline phosphatase (ALP) (U/L)	MS-1-1	14.986 ± 0.294 ^a^	9.01 ± 0.18 ^Ab^	13.89 ± 0.16 ^Ac^
	MS-1-2		8.37 ± 0.19 ^Bb^	13.28 ± 0.172 ^Bc^
	MS-1-3		7.62 ± 0.022 ^Cb^	13.97 ± 0.271 ^Ac^

Note: Dates are expressed as mean ± SD. Means with different lowercase letters indicate a significant difference between time intervals within each sample (*p* < 0.05), while different capital letters indicate significant differences among three treatment samples in each time interval (*p* < 0.05).

**Table 3:** The changes in basic nutrients of the sea bass before and after simulated live transport.

Indicators	Samples	Simulated Transport Time (h)		
		0 (CK)	72	Recover-12
moisture content (%)	MS-1-1	76.68 ± 0.20 ^a^	77.11 ± 0.106 ^Aa^	76.95 ± 0.436 ^Aa^
	MS-1-2		76.69 ± 0.174 ^BCa^	77.25 ± 0.28 ^Aa^
	MS-1-3		76.58 ± 0.212 ^Ca^	76.76 ± 0.28 ^Aa^
Crude fat content (%)	MS-1-1	7.24 ± 0.09 ^a^	4.87 ± 0.11 ^Ab^	2.92 ± 0.06 ^Ac^
	MS-1-2		5.58 ± 0.15 ^Bb^	3.70 ± 0.16 ^Bc^
	MS-1-3		6.23 ± 0.39 ^Bb^	5.29 ± 0.11 ^Cc^
Total protein content (μg/mL)	MS-1-1	573.96 ± 15.16 ^a^	416.94 ± 24.26 ^Ab^	475.82 ± 14.88 ^Ac^
	MS-1-2		479.39 ± 22.39 ^Bb^	481.77 ± 24.91 ^Ab^
	MS-1-3		496.64 ± 15.35 ^Bb^	509.13 ± 8.86 ^Ab^
Muscle glycogen (mg/g)	MS-1-1	3.52 ± 0.03 ^a^	2.44 ± 0.02 ^Ab^	2.66 ± 0.01 ^Ac^
	MS-1-2		2.31 ± 0.01 ^Bb^	2.93 ± 0.01 ^Bc^
	MS-1-3		2.95 ± 0.00 ^Cb^	3.14 ± 0.10 ^Cc^

Note: Dates are expressed as mean ± SD. Means with different lowercase letters indicate a significant difference between time intervals within each sample (*p* < 0.05), while different capital letters indicate significant differences among three treatment samples in each time interval (*p* < 0.05).

**Table 4:** The changes in free amino acid and nucleotide of the sea bass before and after simulated live transport.

Indicators	Samples	Simulated Transport Time (h)		
		0 (CK)	72	Recover-12
Aspartic Acid # (Asp) (mg/kg)	MS-1-1	2.68 ± 0.11 ^a^	2.89 ± 0.16 ^Aa^	2.77 ± 0.06 ^Aa^
	MS-1-2		2.76 ± 0.09 ^Aa^	2.60 ± 0.10 ^Aa^
	MS-1-3		2.83 ± 0.08 ^Aa^	2.79 ± 0.02 ^Aa^
Threonine * (Thr) (mg/kg)	MS-1-1	9.63 ± 0.11 ^a^	11.13 ± 0.05 ^Ab^	9.86 ± 0.11 ^Aab^
	MS-1-2		10.60 ± 0.04 ^Ab^	9.73 ± 0.04 ^Aa^
	MS-1-3		10.29 ± 0.09 ^Aa^	9.70 ± 0.06 ^Aa^
Serine * (Ser) (mg/kg)	MS-1-1	13.60 ± 0.05 ^a^	15.94 ± 0.05 ^Ab^	15.56 ± 0.06 ^Ab^
	MS-1-2		15.77 ± 0.04 ^Bb^	13.50 ± 0.03 ^Aa^
	MS-1-3		14.80 ± 0.00 ^Ba^	13.33 ± 0.25 ^Aa^
Glutamic Acid # (Glu) (mg/kg)	MS-1-1	31.24 ± 0.02 ^a^	35.91 ± 0.04 ^Ab^	33.07 ± 0.02 ^Ac^
	MS-1-2		36.52 ± 0.19 ^Ab^	33.66 ± 0.09 ^Aab^
	MS-1-3		33.93 ± 0.01 ^Ab^	32.81 ± 0.04 ^Ac^
Glycine * (Gly) (mg/kg)	MS-1-1	95.02 ± 0.01 ^a^	99.58 ± 0.01 ^Aa^	97.60 ± 0.07 ^Aa^
	MS-1-2		98.95 ± 0.04 ^Aa^	97.14 ± 0.35 ^Aa^
	MS-1-3		99.33 ± 0.33 ^Aa^	96.40 ± 0.71 ^Aa^
Alanine * (Ala) (mg/kg)	MS-1-1	78.41 ± 0.02 ^a^	91.65 ± 0.04 ^Ab^	81.76 ± 0.50 ^Aa^
	MS-1-2		89.54 ± 0.01 ^Bb^	79.72 ± 0.03 ^Ac^
	MS-1-3		85.78 ± 0.09 ^Cb^	78.55 ± 0.48 ^Aa^
Valine (Val) (mg/kg)	MS-1-1	11.70 ± 0.00 ^a^	13.19 ± 0.08 ^Ab^	12.27 ± 0.00 ^Aab^
	MS-1-2		12.60 ± 0.01 ^Aa^	12.00 ± 0.09 ^Aa^
	MS-1-3		12.81 ± 0.02 ^Aa^	11.93 ± 0.18 ^Aa^
Methionine (Met) (mg/kg)	MS-1-1	6.24 ± 0.00 ^a^	7.86 ± 0.02 ^Ab^	6.66 ± 0.06 ^Aa^
	MS-1-2		7.58 ± 0.66 ^Ab^	6.50 ± 0.06 ^Aab^
	MS-1-3		7.15 ± 0.03 ^Ab^	6.11 ± 0.02 ^Aa^
Isoleucine (Ile) (mg/kg)	MS-1-1	8.03 ± 0.01 ^a^	9.64 ± 0.10 ^Ab^	9.03 ± 0.00 ^Aab^
	MS-1-2		9.23 ± 0.05 ^Ab^	9.08 ± 0.01 ^Ab^
	MS-1-3		9.17 ± 0.00 ^Aa^	9.07 ± 0.13 ^Aa^
Leucine (Leu) (mg/kg)	MS-1-1	14.76 ± 0.01 ^a^	16.95 ± 0.39 ^Aa^	16.08 ± 0.01 ^Aa^
	MS-1-2		16.80 ± 0.00 ^Ab^	15.89 ± 0.01 ^Ac^
	MS-1-3		16.26 ± 0.20 ^Aa^	15.59 ± 0.14 ^Aa^
Tyrosine (Tyr) (mg/kg)	MS-1-1	3.03 ± 0.01 ^a^	5.28 ± 0.02 ^Ab^	3.61 ± 0.00 ^Ac^
	MS-1-2		4.99 ± 0.01 ^Ab^	3.47 ± 0.02 ^Ac^
	MS-1-3		4.08 ± 0.00 ^Bb^	3.19 ± 0.00 ^Bc^
Phenylalanine (Phe) (mg/kg)	MS-1-1	2.93 ± 0.01 ^a^	4.99 ± 0.00 ^Ab^	4.06 ± 0.09 ^Aab^
	MS-1-2		4.79 ± 0.02 ^Ab^	3.36 ± 0.05 ^Aa^
	MS-1-3		3.88 ± 0.01 ^Ba^	3.23 ± 0.06 ^Aa^
Lysine (Lys) #* (mg/kg)	MS-1-1	55.89 ± 0.02 ^a^	86.49 ± 0.19 ^Ab^	73.00 ± 0.16 ^Ac^
	MS-1-2		79.52 ± 0.01 ^Bb^	67.09 ± 0.04 ^Bc^
	MS-1-3		73.79 ± 0.03 ^Cb^	62.28 ± 0.01 ^Cc^
Histidine (His) (mg/kg)	MS-1-1	8.04 ± 0.01 ^a^	13.19 ± 0.00 ^Ab^	10.55 ± 0.01 ^ABc^
	MS-1-2		13.61 ± 0.07 ^Ab^	11.94 ± 0.08 ^Ac^
	MS-1-3		11.74 ± 0.00 ^Bb^	10.04 ± 0.10 ^Bc^
Arginine (Arg) (mg/kg)	MS-1-1	8.50 ± 0.11 ^a^	11.73 ± 0.20 ^Aa^	9.04 ± 0.02 ^Aa^
	MS-1-2		11.70 ± 0.00 ^Ab^	8.65 ± 0.00 ^Aa^
	MS-1-3		10.90 ± 0.10 ^Ab^	8.17 ± 0.07 ^Aa^
Total (mg/kg)	MS-1-1	349.71 ± 5.00 ^a^	422.43 ± 20.19 ^Ab^	383.33 ± 11.66 ^Ac^
	MS-1-2		412.95 ± 6.49 ^Ab^	373.36 ± 10.12 ^Aa^
	MS-1-3		393.58 ± 9.89 ^Ab^	362.60 ± 22.63 ^Aab^
IMP (mg/100g)	MS-1-1	283.44 ± 17.15 ^a^	319.35 ± 4.57 ^Ab^	292.10 ± 6.17 ^Aa^
	MS-1-2		304.14 ± 7.81 ^Ba^	280.84 ± 5.01 ^Aa^
	MS-1-3		299.29 ± 5.27 ^Ba^	285.38 ± 1.77 ^Aa^
AMP (mg/100g)	MS-1-1	26.30 ± 2.16 ^a^	18.19 ± 2.29 ^Ab^	23.34 ± 1.67 ^Aa^
	MS-1-2		19.91 ± 0.65 ^Ab^	24.20 ± 2.27 ^Aab^
	MS-1-3		21.10 ± 0.44 ^Ab^	25.72 ± 1.70 ^Aa^

Note: Taste characteristic for umami marked with #; taste characteristic for sweet marked with *; taste characteristics are unmarked for bitterness. Dates are expressed as mean ± SD. Means with different lowercase letters indicate a significant difference between time intervals within each sample (*p* < 0.05), while different capital letters indicate significant differences among three treatment samples in each time interval (*p* < 0.05).

**Table 5:** The changes in organic acid and minerals of the sea bass before and after simulated live transport.

Indicators	Samples	Simulated Transport Time (h)		
		0 h (CK)	72	Recover-12
Lactic acid (mg/kg)	MS-1-1	161.11 ± 0.96 ^a^	334.49 ± 1.73 ^Ab^	227.64 ± 0.15 ^Ac^
	MS-1-2		300.20 ± 0.56 ^Bb^	224.85 ± 1.02 ^Ac^
	MS-1-3		281.97 ± 0.79 ^Cb^	209.78 ± 2.18 ^Bc^
Acetic acid (mg/kg)	MS-1-1	97.25 ± 1.74 ^a^	141.74 ± 1.54 ^Ab^	128.64 ± 2.15 ^Ac^
	MS-1-2		135.58 ± 1.72 ^Bb^	114.61 ± 4.55 ^Bc^
	MS-1-3		135.34 ± 1.68 ^Bb^	113.42 ± 0.06 ^Bc^
Fumaric acid (mg/kg)	MS-1-1	1.07 ± 0.01 ^a^	1.60 ± 0.01 ^Ab^	1.30 ± 0.00 ^Ac^
	MS-1-2		1.41 ± 0.00 ^Bb^	1.29 ± 0.01 ^ABc^
	MS-1-3		1.30 ± 0.00 ^Cb^	1.26 ± 0.01 ^Bc^
Na (mg/kg)	MS-1-1	480.20 ± 52.09 ^a^	764.98 ± 28.92 ^Ab^	628.77 ± 6.51 ^Ac^
	MS-1-2		821.05 ± 9.97 ^Ab^	783.19 ± 41.69 ^Bb^
	MS-1-3		664.91 ± 123.52 ^Aa^	652.22 ± 28.27 ^Aa^
Mg (mg/kg)	MS-1-1	483.92 ± 35.52 ^a^	602.21 ± 23.97 ^Ab^	561.72 ± 6.61 ^Ab^
	MS-1-2		556.93 ± 42.39 ^Aa^	532.18 ± 19.61 ^Aa^
	MS-1-3		561.01 ± 86.36 ^Aa^	521.93 ± 24.51 ^Aa^
P (mg/kg)	MS-1-1	2729.06 ± 71.26 ^a^	3309.95 ± 190.99 ^Aa^	3054.43 ± 121.93 ^Aa^
	MS-1-2		3214.50 ± 330.87 ^Aa^	2821.68 ± 74.99 ^Aa^
	MS-1-3		2949.47 ± 257.73 ^Aa^	2829.35 ± 97.49 ^Aa^
K (mg/kg)	MS-1-1	7358.79 ± 202.85 ^a^	9260.77 ± 445.85 ^Ab^	8033.81 ± 242.31 ^Aa^
	MS-1-2		8568.97 ± 406.25 ^ABb^	7447.34 ± 256.76 ^ABa^
	MS-1-3		7880.18 ± 504.78 ^Ba^	7410.30 ± 219.16 ^Ba^
Ca (mg/kg)	MS-1-1	60.67 ± 7.80 ^a^	61.38 ± 6.56 ^Aa^	59.31 ± 3.38 ^Aa^
	MS-1-2		60.19 ± 0.73 ^Aa^	60.83 ± 5.91 ^Aa^
	MS-1-3		59.86 ± 8.21 ^Aa^	61.09 ± 1.79 ^Aa^
Fe * (mg/kg)	MS-1-1	15.70 ± 1.91 ^a^	14.06 ± 2.25 ^Aa^	13.45 ± 0.82 ^Aa^
	MS-1-2		15.22 ± 0.79 ^Aa^	19.45 ± 6.70 ^Aa^
	MS-1-3		16.86 ± 1.82 ^Aab^	12.32 ± 0.60 ^Aac^
Cu * (mg/kg)	MS-1-1	4.65 ± 0.02 ^a^	4.40 ± 0.09 ^Aa^	4.49 ± 0.10 ^Aa^
	MS-1-2		4.45 ± 0.09 ^Aa^	4.54 ± 0.07 ^Aa^
	MS-1-3		4.40 ± 0.08 ^Aa^	4.55 ± 0.04 ^Aa^
Zn * (mg/kg)	MS-1-1	8.06 ± 1.47 ^a^	8.80 ± 1.83 ^Aa^	8.44 ± 0.96 ^Aa^
	MS-1-2		8.73 ± 0.80 ^Aa^	8.19 ± 0.81 ^Aa^
	MS-1-3		8.59 ± 1.86 ^Aa^	8.18 ± 1.00 ^Aa^

Note: Dates are expressed as mean ± SD. The asterisk (*) represents trace elements. Means with different lowercase letters indicate a significant difference between time intervals within each sample (*p* < 0.05), while different capital letters indicate significant differences among three treatment samples in each time interval (*p* < 0.05).

## Data Availability

All data, models, and codes generated or used during the study appears in the submitted article.
